# Determinants of Stunting among Children under Five in Pakistan

**DOI:** 10.3390/nu15153480

**Published:** 2023-08-07

**Authors:** Sajid Bashir Soofi, Ahmad Khan, Sumra Kureishy, Imtiaz Hussain, Muhammad Atif Habib, Muhammad Umer, Shabina Ariff, Muhammad Sajid, Arjumand Rizvi, Imran Ahmed, Junaid Iqbal, Khawaja Masuood Ahmed, Abdul Baseer Khan Achakzai, Zulfiqar A. Bhutta

**Affiliations:** 1 Center of Excellence in Women & Child Health, The Aga Khan University, Karachi 74800, Pakistan; sajid.soofi@aku.edu (S.B.S.); ahmad.sherali@aku.edu (A.K.); sumrakureishy@gmail.com (S.K.); imtiaz.hussain@aku.edu (I.H.); habibatif@yahoo.com (M.A.H.); muhammad.umer@aku.edu (M.U.); sajid.muhammad@aku.edu (M.S.); arjumand.rizvi@aku.edu (A.R.); imran.ahmed@aku.edu (I.A.); 2 Department of Pediatrics & Child Health, The Aga Khan University, Karachi 74800, Pakistan; shabina.ariff@aku.edu (S.A.); junaid.iqbal@aku.edu (J.I.); 3 Ministry of Health Services Regulation & Coordination, Islamabad 44020, Pakistan; nfapakistan@gmail.com (K.M.A.); achakzaibk@gmail.com (A.B.K.A.); 4 Lawson Centre for Nutrition, University of Toronto, Toronto, ON M5S 1A8, Canada; 5 Centre for Global Child Health, Hospital for Sick Children, Toronto, ON M5G 1X8, Canada

**Keywords:** undernutrition, childhood stunting, risk factors, children under five, national nutrition survey, Pakistan

## Abstract

Introduction: Child stunting remains a public health concern. It is characterized as poor cognitive and physical development in children due to inadequate nutrition during the first 1000 days of life. Across south Asia, Pakistan has the second-highest prevalence of stunting. This study assessed the most recent nationally representative data, the National Nutrition Survey (NNS) 2018, to identify the stunting prevalence and determinants among Pakistani children under five. Methods: The NNS 2018, a cross-sectional household-level survey, was used to conduct a secondary analysis. Data on malnutrition, dietary practices, and food insecurity were used to identify the prevalence of stunting among children under five years in terms of demographic, socioeconomic, and geographic characteristics. The prevalence of stunting was calculated using the World Health Organization (WHO) height for age z-score references. Univariate and multivariable logistic regressions were conducted to identify the factors associated with child stunting. Results: The analysis showed that out of 52,602 children under five, 40.0% were found to be stunted. Male children living in rural areas were more susceptible to stunting. Furthermore, stunting was more prevalent among children whose mothers had no education, were between 20 and 34, and were employed. In the multivariable logistic regression, male children (AOR = 1.08, 95% CI [1.04–1.14], *p* < 0.001) from rural areas (AOR = 1.07, 95% CI [1.01–1.14], *p* = 0.014), with the presence of diarrhea in the last two weeks (AOR = 1.15, 95% CI [1.06–1.25], *p* < 0.001) and mothers who had no education (AOR = 1.57, 95% CI [1.42–1.73], *p* < 0.001) or lower levels of education (primary: AOR = 1.35, 95% CI [1.21–1.51], *p* < 0.001; middle: AOR = 1.29, 95% CI [1.15–1.45], *p* < 0.001), had higher odds of stunting. Younger children aged < 6 months (AOR = 0.53, 95% CI [0.48–0.58], *p* < 0.001) and 6–23 months (AOR = 0.89, 95% CI [0.84–0.94], *p* < 0.001), with mothers aged 35–49 years (AOR = 0.78, 95% CI [0.66–0.92], *p* = 0.003), had lower odds of stunting. At the household level, the odds of child stunting were higher in lower-income households (AOR = 1.64, 95% CI [1.46–1.83], *p* < 0.001) with ≥ 7 members (AOR = 1.09, 95% CI [1.04–1.15], *p* < 0.001), with no access to improved sanitation facilities (AOR = 1.14, 95% CI [1.06–1.22], *p* < 0.001) and experiencing severe food insecurity (AOR = 1.07, 95% CI [1.01–1.14], *p* = 0.02). Conclusion: Child stunting in Pakistan is strongly associated with various factors, including gender, age, diarrhea, residence, maternal age and education, household size, food and wealth status, and access to sanitation. To address this, interventions must be introduced to make locally available food and nutritious supplements more affordable, improve access to safe water and sanitation, and promote female education for long-term reductions in stunting rates.

## 1. Introduction

Child stunting remains a global public health concern [1,2]. It is characterized as poor cognitive and physical development in children due to inadequate nutrition during the first 1000 days of life—from conception to two years of age [2]. These children cannot attain their full potential and face disadvantages and difficulties in their schooling, careers, and ability to contribute and engage within their communities. In 2020, almost 150 million children under five experienced stunting worldwide [3]. Over the past three decades, the number of children experiencing stunting has decreased by 109 million, with one of the highest reductions observed in south Asia (44 million fewer stunted children from 1990 to 2020) [1]. However, these numbers are expected to increase due to the negative impact of COVID-19 on livelihoods, the affordability of nutritious diets, and access to essential health and nutrition services [4].

The south Asia region comprises seven countries: Afghanistan, Bangladesh, Bhutan, India, Iran, Maldives, Nepal, Pakistan, and Sri Lanka. Regardless of the reduction in the number of stunted children, this region continues to experience a high prevalence of stunting (31.7%), which is significantly higher than the global average (21.3%) [1,3]. Among these seven countries, Pakistan has the second-highest prevalence of stunting after Afghanistan [5,6,7,8,9,10,11,12,13,14]. Pakistan, an agricultural country, has a rapidly growing population of 207 million, headed towards 300 million by 2050 [6,15]. Its population consists of a considerable youth bulge (28% between 15 and 29 years), with 31 million children under five years of age and 56 million women of reproductive age (15–49 years) [6,8]. With a fertility rate of 3.56 children per woman, about 50% of young Pakistani women of reproductive age (15–19 years) leave school, marry, and bear their first child before their 20th birthday [6]. As the main contributor to the rapidly growing population, these young women’s ability to access education, nutritious diets, healthcare services, and maintain adequate birth intervals and water, sanitation, and hygiene practices is a growing concern, as these are strong drivers of healthy child growth [1]. In Pakistan, the failure to tackle the high prevalence of stunting may severely impact economic growth and human capital development, leading to delays in the demographic dividend anticipated for the country [6].

With the country’s efforts towards achieving the 17 Sustainable Development Goals (S.D.G.s), especially S.D.G. 2: Zero Hunger and S.D.G. 3: Good Health and Wellbeing, the national health and nutrition indicators have shown some progress, with stunting prevalence decreasing from 44% to 40% from 2011 to 2018 [6,16]. Similarly, the wasting prevalence (15% in 2011 to 7.1% in 2018), maternal mortality rate (276/100,000 live births in 2007 to 186/100,000 live births in 2019), exclusive breastfeeding rate (37.7% in 2011 to 48.4% in 2018), and child immunization coverage (54% in 2013 to 76.5% in 2021) have improved over the last few years [5,16,17]. No improvements have been made in the anemia rates among women of reproductive age and the prevalence of low birth weight [16]. Against this backdrop, we aimed to assess the most recent nationally representative data, the National Nutrition Survey 2018, to describe the prevalence of stunting in terms of socioeconomic and geographic characteristics. We also aimed to identify the child-, maternal-, and household-level determinants of stunting in Pakistan. These determinants, once identified, may help to improve the understanding and enable the strategic strengthening of nutrition-specific and -sensitive interventions and programs.

## 2. Materials and Methods

The National Nutrition Survey 2018 used a cross-sectional survey design, which collected data at the household level using quantitative and qualitative approaches [6]. In Pakistan, population diversity is entirely hinged on culture, influencing attitudes, practices, political affiliations, and social cohesion [18]. With this in mind, quantitative data were collected at the district level (district representative), while qualitative data were collected at the regional level (regionally representative) [6]. Data on malnutrition, dietary practices (caloric and micronutrient intakes), and food insecurity were collected from all districts of Punjab, Sindh, Khyber Pakhtunkhwa (including erstwhile Federally Administered Tribal Areas (FATA)), Balochistan, Azad Jammu and Kashmir (A.J.K.), Gilgit-Baltistan (G.B.), and Islamabad Capital Territory (I.C.T.). The target population was women of reproductive age (15–49 years), children under five (0–59 months), school-aged children (6–12 years), and adolescents (10–19 years).

The survey used a two-stage stratified sample design, where primary sampling units (P.S.U.s) were provided by the Pakistan Bureau of Statistics based on the Population and Housing Census of 2017 [6]. The households within the P.S.U.s were treated as secondary sampling units (S.S.U.s). Moreover, the prevalence of undernutrition (wasting, stunting, and micronutrient deficiencies) among children under five, adolescents, and women of reproductive age was used to calculate the district-specific sample size. Across the 156 districts of the country, 115,600 households (S.S.U.s) were sampled from 5780 PSUs to obtain reliable estimates of the key survey indicators. A detailed description of the National Nutrition Survey design, sampling methodology, and results are presented elsewhere [6].

The secondary analysis in this article presents the prevalence of stunting among children under five years in terms of maternal age, education, and employment; child gender, age, and presence of disease; and household socioeconomic status and place of residence. Data from 52,602 children under five were used for the secondary analysis. Since stunting was the primary outcome measure, children whose height for the age z-scores was less than -2SD (standard deviation) of the World Health Organization (WHO) Child Growth Standard median were considered to be stunted. The frequencies, along with weighted percentages, were reported for the selected predictors. The analysis started with a simple univariate analysis followed by a multivariate logistic regression. Unadjusted odds ratios with their 95% CIs were reported for the bivariate analysis. Variables significant at *p* < 0.25 were considered for inclusion in the multivariate model. Covariates that were not significant at the multivariate level were dropped consecutively from the model after a careful assessment of confounding. The final model was selected based on the theoretical and statistical significance of the predictors. The Type 1 error was set to 0.05. The model estimates are presented with the adjusted odds ratios (A.O.R.) and 95% CIs.

The analysis was adjusted for the child’s gender, age, and diarrhea in the last two weeks, the mother’s education and age, the family size, sanitation facilities, household food insecurity status, wealth status, rural/urban, and province. All the analyses were performed using Stata statistical software (version 18).

The National Nutrition Survey’s methodology and strategy were approved by the Ethical Review Committee (ERC) at Aga Khan University (A.K.U.) (5176-WCH-ERC-17, dated 27 December 2017). Ethical clearance was obtained from the National Bioethics Committee (N.B.C.) (NBC-278, dated 7 November 2017). Informed consent was obtained from all the participants and confidentially was ensured as part of the survey. Approval for the secondary data analysis was obtained from the ERC, A.K.U.

## 3. Results

### 3.1. Child, Maternal, and Household Characteristics

Out of the 52,602 children enrolled, 50.7% were male, with the majority aged 24–59 months (63.0%) and living in rural areas (63.4%) (Table 1). Across the provinces, most children were from Punjab (52.8%), 27.9% were from Sindh, and 0.5% were from Gilgit Baltistan. A larger proportion of children had no reported presence of diarrhea (90.7%), acute respiratory infection (97.7%), or fever (85.5%) in the last two weeks. More than ½ of the mothers of the children had no education (55.3%), while a ¼ had completed secondary (12.3%) or higher education (11.1%). Most of these women were aged 20–34 (75.0%) and were housewives (89.4%). Most households comprised ≤6 family members (51.7%), had <5 children under the age of five (98.0%), and had access to improved drinking water (92.0%) and improved sanitation facilities (82.5%). Furthermore, 58.4% of households reported being food secure, with 20.2% reporting severe food insecurity. In total, 6 out of 100 households (5.5%) reported receiving financial assistance from the government in the last 12 months. A similar proportion of children (about 20%) were found across the five wealth quintiles, with a 2.1% increase in the poorest quintile.

### 3.2. Determinants of Stunting in Children under Five

Of the 52,602 children under five enrolled in the survey, 40.0% were stunted. In the univariate logistic analysis, the odds of stunting were higher among male children (OR = 1.08, 95% CI [1.03–1.13], *p* = 0.001) from rural areas (OR = 1.43, 95% CI [1.36–1.50], *p* < 0.001), with the presence of diarrhea (OR = 1.26, 95% CI [1.16–1.36], *p* < 0.001), respiratory infection (OR = 1.21, 95% CI [1.06–1.38], *p* = 0.005), or fever (OR = 1.08, 95% CI [1.01–1.15] *p* = 0.017) in the last two weeks compared to female children from urban areas with no diarrhea, respiratory infection, or fever (Table 2). Children under two years of age (<6 months: OR = 0.54, 95% CI [0.50–0.59], *p* < 0.001; 6–23 months: OR = 0.87, 95% CI [0.83–0.92], *p* < 0.001) had lower odds of stunting compared to children aged 24–59 months. Compared to children whose mothers had completed higher education, the odds of stunting were higher among children born to mothers with no education (OR = 2.30, 95% CI [2.11–2.51], *p* < 0.001) or lower levels of education (primary: OR = 1.62, 95% CI [1.46–1.80], *p* < 0.001; middle: OR = 1.45, 95% CI [1.29–1.62], *p* < 0.001; and secondary: OR = 1.17, 95% CI [1.05–1.30], *p* = 0.004). Children with employed mothers (OR = 1.13, 95% CI [1.05–1.21], *p* < 0.001) had higher odds of stunting compared to children whose mothers were housewives. Expectedly, the odds of child stunting decreased with an increase in the mother’s age; however, the odds ratios were not statistically significant for mothers aged 20–34 years (OR =0.88 (95% CI [0.76–1.03], *p* = 0.11) and 35–49 years (OR = 0.91, 95% CI [0.78–1.07], *p* = 0.26), relative to mothers < 20 years of age. The odds of child stunting were higher in households with ≥7 members (OR = 1.08, 95% CI [1.01–1.15], *p* = 0.01), with no access to improved sources of drinking water (OR = 1.15, 95% CI [1.06–1.26], *p* = 0.001) and no improved sanitation facilities (OR = 1.79, 95% CI [1.69–1.89], *p* < 0.001), compared to households with ≤6 members and access to improved drinking water and sanitation facilities. The odds of stunting increased with the severity of food insecurity experienced by households. Households experiencing severe food insecurity (OR = 1.52, 95% CI [1.43–1.61], *p* < 0.001) were most at risk compared to food-secure households. Like food insecurity, the odds of child stunting increased as the wealth status of households decreased. Across the wealth quintiles, the lowest-income households (OR = 2.65, 95% CI [2.45–2.87], *p* < 0.001) were most at risk of experiencing child stunting compared to the wealthiest households. However, unexpectedly, the odds of stunting were lower among children whose households did not receive financial assistance in the last 12 months (OR = 0.62, 95% CI [0.56–0.68], *p* < 0.001) and were located in Punjab (OR = 0.64, 95% CI [0.59–0.70], *p* < 0.001), K.P. (OR = 0.733, 95% CI [0.66–0.81], *p* < 0.001), I.C.T. (OR = 0.54, 95% CI [0.45–0.65], *p* < 0.001), and A.J.K. (OR = 0.74, 95% CI [0.66–0.84], *p* < 0.001) compared to households who received assistance and were located in G.B.

In the multivariable logistic regression, male children (AOR = 1.08, 95% CI [1.04–1.14], *p* < 0.001) from rural areas (AOR = 1.07, 95% CI [1.01–1.14], *p* = 0.014), with the presence of diarrhea in the last two weeks (AOR = 1.15, 95% CI [1.06–1.25], *p* < 0.001), remained at risk of stunting compared to female children from urban areas with no diarrhea in the last two weeks. Younger children aged < 6 months (AOR = 0.53, 95% CI [0.48–0.58], *p* < 0.001) and 6–23 months (AOR = 0.89, 95% CI [0.84–0.94], *p* < 0.001) continued to experience lower odds of stunting as compared to older children aged 24–59 months. The relationship between the risk of child stunting and maternal education remained apparent; however, the odds ratios decreased with no maternal education (AOR = 1.57, 95% CI [1.42–1.73], *p* < 0.001) or lower levels of maternal education (primary: AOR = 1.35, 95% CI [1.21–1.51], *p* < 0.001; middle: AOR = 1.29, 95% CI [1.15–1.45], *p* < 0.001). The odds of stunting were lower among children whose mothers were 35–49 years old (AOR = 0.78, 95% CI [0.66–0.92], *p* = 0.003) compared to mothers aged < 20 years old, which was initially found to be insignificant in the univariate analysis. At the household level, the risk of child stunting remained higher in households with ≥ 7 members (AOR = 1.09, 95% CI [1.04–1.15], *p* < 0.001), with no access to improved sanitation facilities (AOR = 1.14, 95% CI [1.06–1.22], *p* < 0.001) and experiencing severe food insecurity (AOR = 1.07, 95% CI [1.01–1.14], *p* = 0.02) compared to households with ≤ 6 members, access to improved sanitation facilities, and food security. Similarly, the lowest-income households (AOR = 1.64, 95% CI [1.46–1.83], *p* < 0.001) remained most at risk of experiencing child stunting, with this risk gradually decreasing across the wealth quintiles relative to the wealthiest households. Households located in Punjab (AOR = 0.79, 95% CI [0.72–0.87], *p* < 0.001), K.P. (AOR = 0.74, 95% CI [0.67–0.83], *p* < 0.001), and I.C.T. (AOR = 0.80, 95% CI [0.66–0.98], *p* = 0.032) continued to experience higher odds of child stunting, with the new inclusion of Balochistan (AOR = 0.87, 95% CI [0.78–0.98], *p* = 0.022) and FATA (AOR = 0.71, 95% CI [0.59–0. 85], *p* < 0.001), compared to households located in G.B.

## 4. Discussion

The prevalence of child stunting in Pakistan remains very high (44%) compared to the regional (31.7%) and global (21.3%) estimates [6]. This national stunting prevalence is only surpassed by Afghanistan, a country that has been experiencing protracted conflict for over 20 years [5,6,7,8,9,10,11,12,13,14]. Our study presents selected determinants associated with stunting among children under five in Pakistan using the National Nutrition Survey 2018. The study showed that a child’s gender, age, presence of diarrhea, and place of residence, the mother’s age and education, the household size, food security status, wealth status, and access to sanitation facilities were significantly associated with child stunting. Among these determinants, household wealth status and maternal education were most significantly associated with stunting in children under five. Children in the lowest-income households had the highest odds of being stunted, which could be linked to their inability to access safe, diverse, affordable, and nutritious foods and essential health services. This makes these households more vulnerable to food insecurity and poor child growth [19,20,21]. Similar findings have been reported by other studies within the region [19,20,21].

Our analysis found that children living in rural areas were more at risk of stunting, which is consistent with regional surveys conducted in Bangladesh, Nepal, India, and the Maldives [19,20,21]. However, the most recent National Demographic and Health Survey conducted in 2017 found that children living in urban areas had higher odds of stunting; this may be partly due to the rapidly growing population being accompanied by an even faster rate of urbanization [5,22]. One third of the Pakistani population live in urban areas [23]. This urban transition is expected to continue and brings about informal settlements, where the living conditions may be unsafe and access to affordable, nutritious food and essential services, such as water, electricity, sanitation, and health, is very difficult [24,25,26].

Moreover, male children aged 24–59 months were more at risk of stunting than those younger (<24 months) and female. This is consistent with previous research studies, which have shown that male children are most at risk of stunting. In contrast, older children were at risk due to the inappropriate initiation of complementary feeding practices. [19,20,21]. The study also found that children whose mothers had no education and were younger (<20 years) had a higher risk of stunting than children of older mothers with a higher education. Several studies have found a similar relationship between child stunting and maternal education and age [19,20,21]. Older, educated mothers are assumed to be better informed and, therefore, able to respond better and attain their children’s essential nutrition and health requirements [21].

With an estimated 10.8 billion USD already invested in nutrition-specific and health-system-geared interventions over the past decade, the study findings indicate the need for scaling up nutrition-sensitive interventions, which focus on improving the affordability of locally available nutritious foods, access to safe, clean water and sanitation, and encouraging female education, in order to tackle child stunting in Pakistan [27]. Policies and programs focused on social protection, food, education, and water, sanitation, and hygiene systems are critical for positive nutrition and health outcomes, female secondary education attainment, and the transformation of local food systems.

Social protection consists of government policies and programs that prevent and protect people from poverty, vulnerability, and social exclusion throughout their lifecycle [28]. These programs can improve the access to essential services, reduce negative coping strategies in response to shocks, and improve the accessibility and affordability of nutritious foods. Social protection is more effective and creates more remarkable change when food and nutrition security are integrated into national policies and programs. At the same time, the targeting of beneficiaries and the amounts and modalities of transfers are adapted to the primary and nutritional needs of the most vulnerable populations [29].

In Pakistan, efforts are underway to address child stunting by scaling up nutrition-sensitive social protection programs that integrate food security and nutrition outcomes and target the most nutritionally vulnerable populations (women and children). In this context, and with World Bank funds, the government has implemented the Nashonuma project (2020–2023) [30]. This project focuses on demand-side interventions delivered through national health and social protection systems. These interventions include providing conditional cash transfers upon the consumption of locally produced specialized nutritious foods (a lipid-based nutrient supplement), the uptake of maternal and child health services, and maternal participation in social and behavioral change communication activities, in order to improve dietary and hygiene practices and prevent stunting in children. However, there is a lack of supply-side interventions across the food system, which positively influence the food production, processing, availability, and affordability of locally produced nutritious foods—a critical approach to achieving nutritious diets for all.

Our study also has some strengths and limitations. The strengths include using the most recent and largest representative sample, with a high nationwide response rate (90%). In contrast, using a cross-sectional survey design is a limitation, since it prevents the establishment of a causal relationship between child stunting and the different determinants.

## 5. Conclusions

In conclusion, our study found that a child’s gender, age, the presence of diarrhea, and place of residence, the mother’s age and education, the household size, food security status, wealth status, and access to sanitation facilities are significantly associated with child stunting in Pakistan. These factors need an in-depth qualitative investigation in future studies, targeting the rural areas of the country, where the child stunting prevalence is more pronounced. Moreover, the establishment of causal relationships between child stunting and these various determinants would benefit from longitudinal studies. By observing and tracking these factors over time, research studies can provide insights into the true causal influences, enabling more informed decision making and targeted policy interventions. Furthermore, there is a need to scale up nutrition-sensitive interventions focused on improving the affordability of locally available nutritious foods and supplementation, access to safe, clean water and sanitation, and encouraging female education to sustain reduced child stunting in the country.

## Figures and Tables

**Table 1:** Child, maternal, household, and community characteristics among children aged 0–59 months from the NNS 2018 (*N* = 52,602).

Maternal, Child, Household, and Community Characteristics	n (%)
**Maternal Characteristics**	
Mother’s education	
None	30,883 (55.3)
Primary	5639 (12.1)
Middle	4671 (9.2)
Secondary	5836 (12.3)
Higher	5573 (11.1)
Maternal working status	
Housewife	46,344 (89.4)
Others	6258 (10.6)
Mother’s age	
Less than 20 years	1047 (1.9)
20–34 years	38,368 (75.0)
35–49 years	13,187 (23.0)
**Child Characteristics**	
Gender	
Male	26,826 (50.7)
Female	25,776 (49.3)
Child’s age	
<6 months	4388 (8.7)
6–23 months	14,618 (28.3)
24–59 months	33,596 (63.0)
Diarrhea in the last 2 weeks	
Yes	5071 (9.3)
No	47,531 (90.7)
A.R.I. in the last 2 weeks	
Yes	1652 (2.3)
No	50,950 (97.7)
Fever in the last 2 weeks	
Yes	8043 (14.5)
No	44,559 (85.5)
**Household Characteristics**	
Family size	
≤6 members	26,054 (51.7)
7 or more members	26,548 (48.3)
Number of children under five	
<5	51,612 (98.0)
≥5	990 (2.0)
Drinking water sources	
Improved sources	47,203 (92.0)
Unimproved sources	5399 (8.0)
Sanitation facilities	
Improved sanitation facility	41,341 (82.5)
Unimproved sanitation facility	11,261 (17.5)
Food insecurity status	
Food secure	30,741 (58.4)
Mildly food insecure	6157 (12.5)
Moderately food insecure	4337 (8.9)
Severely food insecure	11,367 (20.2)
The household received financial assistance in the last 12 months	
Yes	2853 (5.5)
No	49,749 (94.5)
Wealth status (quintiles)	
Lowest income	14,682 (22.1)
Second	12,152 (20.2)
Middle	10,435 (20.2)
Fourth	8817 (20.0)
Highest income	6516 (17.5)
**Community Characteristics**	
Area	
Urban	15,497 (36.6)
Rural	37,105 (63.4)
Province	
Punjab	19,396 (52.8)
Sindh	10,643 (27.9)
KP	6136 (9.6)
Balochistan	7849 (5.4)
ICT	719 (1.1)
FATA	1043 (1.0)
AJK	3646 (1.6)
GB	3170 (0.5)

Abbreviations: A.R.I., acute respiratory infection; K.P., Khyber Pakhtunkhwa; I.C.T., Islamabad Capital Territory; FATA, erstwhile Federally Administered Tribal Areas; A.J.K., Azad Jammu and Kashmir; and G.B., Gilgit-Baltistan.

**Table 2:** Determinants of stunting among children aged 0–59 months in the NNS 2018 (N = 52,602).

Characteristics	Stunted	Normal	Unadjusted Odd Ratio (OR) [95%CI]	*p*-Values	Adjusted Odd Ratio (OR) [95%CI]	*p*-Values
**Overall**	22,005 (40.0%)	30,597 (60.0%)				
**Maternal Characteristics**						
Mother’s education						
None	14,517 (46.1%)	16,366 (53.9%)	2.308 (2.116–2.518)	<0.001	1.571 (1.423–1.735)	<0.001
Primary	2193 (37.6%)	3446 (62.4%)	1.626 (1.462–1.809)	<0.001	1.358 (1.215–1.518)	<0.001
Middle	1730 (35.0%)	2941 (65.0%)	1.45 (1.295–1.623)	<0.001	1.293 (1.151–1.453)	<0.001
Secondary	1914 (30.3%)	3922 (69.7%)	1.174 (1.053–1.308)	0.004	1.109 (0.993–1.239)	0.066
Higher	1651 (27.0%)	3922 (73.0%)	Ref.		Ref.	
Maternal working status						
Housewife	19,316 (39.7%)	27,028 (60.3%)	Ref.			
Others	2689 (42.7%)	3569 (57.3%)	1.134 (1.058–1.215)	<0.001		
Mother’s age						
Less than 20 years	456 (42.7%)	591 (57.3%)	Ref.		Ref.	
20–34 years	15,997 (39.8%)	22,371 (60.2%)	0.885 (0.76–1.032)	0.119	0.895 (0.765–1.048)	0.168
35–49 years	5552 (40.6%)	7635 (59.4%)	0.914 (0.781–1.07)	0.264	0.783 (0.665–0.922)	0.003
**Child Characteristics**						
Gender						
Male	11,520 (41.0%)	15,306 (59.0%)	1.083 (1.035–1.133)	0.001	1.089 (1.04–1.141)	<0.001
Female	10,485 (39.0%)	15,291 (61.0%)	Ref.		Ref.	
Child’s age						
<6 months	1360 (28.4%)	3028 (71.6%)	0.545 (0.5–0.595)	<0.001	0.534 (0.488–0.585)	<0.001
6–23 months	5899 (38.9%)	8719 (61.1%)	0.874 (0.83–0.921)	<0.001	0.893 (0.847–0.942)	<0.001
24–59 months	14,746 (42.1%)	18,850 (57.9%)	Ref.		Ref.	
Diarrhea in the last 2 weeks						
Yes	2366 (45.2%)	2705 (54.8%)	1.262 (1.169–1.363)	<0.001	1.155 (1.067–1.25)	<0.001
No	19,639 (39.5%)	27,892 (60.5%)	Ref.		Ref.	
ARI. in the last 2 weeks						
Yes	778 (44.6%)	874 (55.4%)	1.211 (1.061–1.383)	0.005		
No	21,227 (39.9%)	29,723 (60.1%)	Ref.			
Fever in last 2 weeks						
Yes	3533 (41.6%)	4510 (58.4%)	1.081 (1.014–1.151)	0.017		
No	18,472 (39.7%)	26,087 (60.3%)	Ref.			
**Household Characteristics**						
**Family Size**						
≤6 members	10,679 (39.1%)	15,375 (60.9%)	Ref.		Ref.	
7 or more members	11,326 (41.0%)	15,222 (59.0%)	1.081 (1.014–1.151)	0.017	1.098 (1.048–1.151)	<0.001
**Number of Children Under Five**						
<5	21,567 (40.0%)	30,045 (60.0%)	Ref.			
≥5	438 (39.6%)	552 (60.4%)	0.981 (0.834–1.154)	0.817		
**Drinking Water Sources**						
Improved sources	19,505 (39.7%)	27,698 (60.3%)	Ref.			
Unimproved sources	2500 (43.3%)	2899 (56.7%)	1.158 (1.062–1.262)	0.001		
**Sanitation Facilities**						
Improved sanitation facility	16,276 (37.5%)	25,065 (62.5%)	Ref.		Ref.	
Unimproved sanitation facility	5729 (51.9%)	5532 (48.1%)	1.796 (1.699–1.898)	<0.001	1.144 (1.068–1.226)	<0.001
**Food Insecurity Status**						
Food secure	11,930 (36.7%)	18,811 (63.3%)	Ref.		Ref.	
Mildly food insecure	2670 (41.6%)	3487 (58.4%)	1.225 (1.14–1.317)	<0.001	1.099 (1.021–1.182)	0.012
Moderately food insecure	1986 (43.8%)	2351 (56.2%)	1.343 (1.231–1.465)	<0.001	1.057 (0.968–1.156)	0.217
Severely food insecure	5419 (46.9%)	5948 (53.1%)	1.523 (1.439–1.612)	<0.001	1.078 (1.012–1.149)	0.020
**The household received financial assistance in the last 12 months**						
Yes	1402 (51.0%)	1451 (49.0%)	Ref.			
No	20,603 (39.4%)	29,146 (60.6%)	0.625 (0.569–0.687)	<0.001		
**Wealth Status (quintiles)**						
Lowest income	7552 (51.9%)	7130 (48.1%)	2.659 (2.455–2.879)	<0.001	1.64 (1.468–1.832)	<0.001
Second	5429 (45.0%)	6723 (55.0%)	2.019 (1.861–2.191)	<0.001	1.477 (1.341–1.627)	<0.001
Middle	4124 (39.6%)	6311 (60.4%)	1.612 (1.483–1.752)	<0.001	1.285 (1.172–1.409)	<0.001
Fourth	2972 (32.3%)	5845 (67.7%)	1.177 (1.079–1.283)	<0.001	1.035 (0.946–1.132)	0.452
Highest income	1928 (28.9%)	4588 (71.1%)	Ref.		Ref.	
**Community Characteristics**						
**Area**						
Urban	5714 (34.7%)	9783 (65.3%)	Ref.		Ref.	
Rural	16,291 (43.2%)	20,814 (56.8%)	1.43 (1.36–1.504)	<0.001	1.078 (1.016–1.144)	0.014
**Province**						
Punjab	7146 (36.5%)	12,250 (63.5%)	0.649 (0.594–0.709)	<0.001	0.799 (0.727–0.879)	<0.001
Sindh	4904 (45.7%)	5739 (54.3%)	0.95 (0.866–1.042)	0.273	0.942 (0.852–1.041)	0.241
KP	2536 (39.4%)	3600 (60.6%)	0.733 (0.662–0.813)	<0.001	0.748 (0.672–0.833)	<0.001
Balochistan	3870 (46.8%)	3979 (53.2%)	0.992 (0.894–1.101)	0.887	0.878 (0.785–0.982)	0.022
ICT	235 (32.6%)	484 (67.4%)	0.545 (0.452–0.658)	<0.001	0.808 (0.665–0.982)	0.032
FATA	463 (44.7%)	580 (55.3%)	0.911 (0.766–1.084)	0.294	0.717 (0.599–0.858)	<0.001
AJK	1395 (39.8%)	2251 (60.2%)	0.747 (0.663–0.841)	<0.001	0.91 (0.803–1.032)	0.142
GB	1456 (47.0%)	1714 (53.0%)	Ref.		Ref.	

Abbreviations: CI; confidence interval; ARI, acute respiratory infection; KP, Khyber Pakhtunkhwa; ICT, Islamabad Capital Territory; FATA, erstwhile Federally Administered Tribal Areas; AJK, Azad Jammu and Kashmir; and GB, Gilgit-Baltistan.

## Data Availability

The data presented in this study are available on request from the corresponding author.
